# Toxicological impacts of biogenic zinc oxide nanoparticles on blue Parrotfish using multibiomarker assessment

**DOI:** 10.1038/s41598-026-36870-y

**Published:** 2026-02-15

**Authors:** Ahmed E. Alprol, Tarek M. Hamad, Hossam E. R. Sharaf, Heba Saad El-Sayed, Khouloud M. Barakat, Ahmed Abouelwafa, Hanan M. Khairy

**Affiliations:** National Institute of Oceanography and Fisheries, NIOF, Cairo, Egypt

**Keywords:** Zinc oxide nanoparticles, Scarus coeruleus, Oxidative stress, Liver enzymes, Ionic imbalance, Nanotoxicity, Padina pavonica, Histopathology, Microbiology, Biochemistry, Environmental sciences, Microbiology

## Abstract

This research demonstrates how using *Padina pavonica* extract to create zinc oxide nanoparticles (ZnO-NPs) can be toxic to aquatic organisms. Blue Parrotfish (*Scarus coeruleus*) were used to evaluate the effects of ZnO-NPs over a 15-day period at different concentration levels (0–80 mg/L). The mortality rate was closely related to the concentrations tested; therefore, at 80 mg/L, every single fish died from exposure. The indication of oxidative stress through the decrease of glutathione (GSH) levels, and a nonlinear relationship of the reactive oxygen species (ROS) suggests cellular adaptation occurred during this time. As expected, there was also an overall decrease in activity of hepatic enzymes (ALT, AST, ALP) due to damage done to the liver; this was supported further through the tissue analysis by histopathology assessment. Additionally, the ionic homeostasis is disrupted by increased tissue levels of sodium (Na^+^), potassium (K^+^), and calcium (Ca²^+^). At sublethal levels, ZnO-NPs also inhibited bacterial growth (*Streptococcus* and *Vibrio*), demonstrating both toxic and antibacterial properties These findings highlight the ecological risks associated with nanoparticle-driven marine pollution and emphasize the need to establish environmentally safe exposure limits to protect coastal and marine ecosystems.

##  Introduction

 Modern science and technology have made it possible to identify and use certain technologies, leading to one more source of new pollution: the continuous discharge of diverse toxic substances into the natural environment and the attendant threat to ecological balance and human health^1^. The rapid expansion of applications of nanoparticles into everyday life and industrial sectors has added an extra dimension governed by the progress of science and technology^2^. Nanotechnology revolves around the production and utilization of nanomaterials, many of which are now utilized globally for their antibacterial, antifungal, anti-inflammatory, and wound-healing properties^3^. Nanomaterials bear unique lifelikeness features that set them apart from their bulks. Considerable proportion of atoms are on the outer side leading to their increased reactivity^4^. Metal oxide nanoparticles are ubiquitous in commercial products, thus posing potential environmental health concerns^5^. They have many benefits, for example, remediation of toxic dyes in industrial wastewater^6^. In industry, zinc oxide nanoparticles (ZnO-NPs) are applied in catalysts, gas sensors, biomedicine, and environmental remediation^7^. Owing to their distinctive properties and various structures, ZnO-NPs are widely employed in optoelectronics, ceramics, cosmetics, catalysis, and pigments^8^.

The very same perspective would also hold for those species that behave better when making near distances with the electrodes. These fish species would be from the genera of carp and tilapia. Bass, salmon, and trout, since they are the best predators currently expert in their driving distance hunting behaviors, would have no kind of special orientation preference within the pH salinity range being mentioned. With the objective of understanding the underlying reasons why those species would combine more easily and effectively, it could be narrowing the focus on the possible aquatic habitat drivers: their inhabiting different types of habitats at different distances away from the electrodes^9^. These fish feed very close to the electrodes, and some prefer to feed at specific pH or salinity ranges. This list would restrict the possible habitats in which those species might combine to a very select few, remarkably one or two among diverse categories of fish^10^. Now, since mound stream trout are fine predators, and their forest habitats take them hunting for prey wherever placed along the boundary of pH salinity, one cannot enforce preference for a specific orientation along pH salinity either. It would be quite interesting to work with something like this so as to get at the question of why particular species allow mingling so readily and totally, which would seem to narrow down possible forces of a few others-their blissfully cohabiting specific distances from the electrodes, with shared attributes like little difference in oxygen levels, or perhaps low salinity as well.

Extremely dispersed, at least earlier on in their early lifespan, ZnO-NPs presently rank third in global demand and usage of nanomaterials following titanium oxide and silicon oxide^11^. They are therefore highly desirable for applications in broadband semiconductors, several catalytic systems, gas sensing, and microelectronics development^12^. However, nowadays, their extensive use is becoming problematic for aquatic ecosystems as these nano-silvers can enter water bodies through wastewater discharge. Because of potent and toxic to aquatic organisms, ZnO-NPs inspired significant research considerations^13^.

The high solubility of ZnO-NPs in water enhances their toxicity towards aquatic organisms^14^. They are absorbed by aquatic organisms at 15–20 times higher rates than that of bulk counterparts^15^. Resistance towards bioaccumulation; moreover, greater cellular responses to ZnO-NPs versus bulk ZnO have been noticed in experiments performed on young Common Carp (*Cyprinus carpio*)^16^. Although sometimes included in fish feed as supplements, the introduction of ZnO-NPs into aquatic environments has been shown to be toxic through changes in serum proteome profiles^17^. Gills, stomach, and liver bioaccumulation, together with increased lipid peroxidation, have been reported^17^. Reactive oxygen species (ROS) may be produced due to ZnO-NPs exposure, giving rise to DNA injury. Hatching is delayed due to oxidative stress induced by ZnO-NPs exposure to zebrafish (Danio rerio) embryos^18^.

Other studies showed the subchronic exposure to ZnO-NPs produced histopathological and serum biochemical changes in O. niloticus^19^. Aside from environmental reasons, ZnO-NP could be hazardous to human health. Studies show that exposure to these nanoparticles can affect pulmonary histology^20^. The increasing proof of their effect reflects the need for further research to determine their safe and responsible use.

The growing release of these materials into aquatic systems brings environmental and biological risks. In this regard, this study will examine the toxicological effects of green-synthesized ZnO-NPs on the Blue Parrotfish (*Scarus coeruleus*), focusing on oxidative stress markers, liver enzyme activities, and mineral composition in muscle tissues. Parrotfish are vibrant tropical creatures that dedicate nearly 90% of their time to feeding on algae found on coral reefs. Their continuous feeding plays a crucial role in maintaining the cleanliness of the reefs, which supports the health and growth of corals^21^.

As aquatic organisms are very sensitive, such studies would assess the relevance of ZnO-NPs on fish health as possible and ecological toxicological risk. So far, various studies have reported on the toxicity of ZnO-NPs on fish species, but really little information exists on toxicological effects in Blue Parrotfish. This study offers valuable insights into the biochemical and physiological responses of this species to ZnO-NPs exposure, thus filling a major gap in aquatic nanotoxicology.

Therefore, the research objectives are determining zinc concentrations dissolved, mortality rate, and lethality to fish. The concentration of ZnO-NPs should be correlated with the amount of dissolving zinc ions into the water with respect to the mortality rate of fishes. This relationship enables these dynamics to be determined for rectifying acute toxicity and establishing safe exposure levels. Analyze oxidative stress and antioxidant responses by measuring oxidative stress biomarkers such as reactive oxygen species (ROS), superoxide dismutase (SOD), catalase (CAT), glutathione peroxidase (GPx), and malondialdehyde (MDA) in liver and gill tissues, this study explores the role of ZnO-NPs in oxidative damage and antioxidant defense mechanisms. Assess liver enzyme activities, changes in aspartate aminotransferase (AST) and alanine aminotransferase (ALT) levels provide insights into liver function and potential hepatotoxicity caused by ZnO-NPs exposure. Determine mineral composition in muscle tissues by the study evaluates the effects of ZnO-NPs on essential mineral levels (sodium, calcium, and potassium) in fish muscle, which are crucial for maintaining physiological homeostasis. Despite increasing awareness of nanomaterial toxicity in aquatic environments, there is limited knowledge on the biochemical and physiological responses of Blue Parrotfish to ZnO-NPs exposure. Most existing studies focus on conventional heavy metal toxicity, while the distinct mechanisms associated with nanoparticle interactions remain underexplored. This study bridges that gap by integrating biochemical assays, enzymatic activities, and mineral composition analysis to provide a holistic understanding of ZnO-NPs toxicity in marine fish.

##  Materials and methods

### Reagents and materials

Brown algae *Padina pavonica* were collected from Ras Sidr in Egypt along the Red Sea coast. Zinc nitrate hexahydrate (Zn(NO_3_)_2_.6H_2_O) of purity 99.9% was obtained from Sigma Aldrich. Methylene blue dye (purity ≥ 99.0%) was sourced from Novia Hexachem. Sodium hydroxide and hydrochloric acid were used to adjust the pH (NaOH: 98%; HCl: 37%). All chemicals were of analytical grade, and no further purification was needed.

### Green synthesis of ZnO nanoparticles using *Padina Pavonica* extract

The use of biological materials such as algal extracts in nanoparticle synthesis provides an eco-friendly alternative to classical chemical methods. This approach reduces the use of hazardous chemicals and extreme conditions, thus serving as a more sustainable approach for nanoparticle production.

- Algae Preparation.

*Padina pavonica*, a marine algae species known for its bioactive compounds, was selected as a natural source for synthesis. The algae were thoroughly rinsed with distilled water to get rid of sand, debris, and any other impurities. Sanitizing this portion prevents contamination of the very end products. The cleaned algae were then dried in an oven at 60 °C for 24 h, this temperature was adequate to eliminate moisture without altering the bioactive components. After being dried, the powder was further ground using a mixer to further increase the surface area and, in turn, the efficiency of the extraction.

b) Algae Extract Preparation.

In total, 10 g of powdered algae mixed into 100 ml of distilled water were boiled for 30 min to help release bioactive compounds. The period of boiling was selected to ensure maximum possible extraction without altering the effectiveness of the active part. The extract was filtered through Whatman No. 1 filter paper to remove undissolved particles; this yielded essentially a clear solution. The extract was stored at temperatures of 10 °C to maintain its stability against microbial attack.

c) Synthesis of ZnO Nanoparticles.

The nanoparticles of ZnO were synthesized by using the co-precipitation method. The algae extract was mixed with 50 mL of 0.1 M zinc nitrate hexahydrate solution while the mixing lasted, which would help in starting the reaction of zinc ions with bioactive compounds. The reaction mixture was made alkaline to a pH of 10 using 0.1 M sodium hydroxide. The solution was then kept at room temperature (~ 25 °C) for 2 h for precipitation to take place completely. The precipitate containing the precursor for zinc oxide was collected by centrifugal force at 5000 rpm for 10 min. It was then washed several times with distilled water to remove any remaining impurities. The material was dried at 80 °C for 12 h to remove excess moisture. Calcination was done at 300 °C for 2 h for finalizing the formation of the ZnO nanoparticles.

### Characterization of synthesized ZnO nanoparticles

Several characterization techniques were employed to assess synthesized ZnO nanoparticles for the evaluation of their physicochemical features. Identification of the functional groups present in the nanocomposite was achieved using Fourier-transform infrared (FTIR) spectroscopy in the spectral range of 4000 to 400 cm⁻¹. Zeta potential measurement was carried out at 25.0 °C with 20 runs of 34.6 kcps recorded on a disposable zeta cell at a measurement distance of 2.00 mm. Energy Dispersive X-ray Spectroscopy (EDX) was executed to examine the elemental composition by Scanning Electron Microscopy (SEM). X-ray powder diffraction analyses with CuKα radiation (40 kV, step size 0.02, scan rate 0.5 min⁻¹, 2θ range 20–80˚) have mainly been used to establish the crystalline phases of the synthesized nanoparticles.

### Experimental fish

The fish samples for the experiment and water analysis were obtained from Marine hatchery located in El- Max research station NIOf-Ornamental fish production unit. Thirty six Blue parrotfish fingerlings (*Scarus coeruleus*), weighing an average, were used for each nanomaterial concentration in duplicated trial. Acclimatization was performed in a 2-liter tank having fresh water for a period of 2 weeks. After that, the fish were separated into six groups with each of them exposed to increasingly higher concentrations of ZnO-NPs at 0, 10, 20, 40, 60, and 80 mg/L. A control group was set up in which no nanoparticles were added to the tanks for comparison purposes. The exposure duration to the ZnO-NPs lasted a total of 15 days, beginning on the first day of the experiment. During this time, fish were provided with commercial feed (C-5003, Uni-President, Di An, Vietnam) at a rate equal to 3% of their body weight. Basic water quality parameters, including pH, dissolved oxygen (DO), total ammonia, nitrite, and nitrate, were closely monitored throughout the exposure period. The experiment was conducted in 12 glass containers, each containing 2 L of fresh water. There were three fish kept in each tank all under conditioned settings as described above. The concentration of ZnO-NPs was monitored continuously in the water to check for any eventual decrease of concentration. On the contrary, the first concentration of the nanoparticles was not sometimes replaced; and replenishment occurred shortly after the respective concentration was diminished by necessity. Finally, we use clove oil as anesthetic agent with concentration 0.05 ml per liter of water to calm the fish when taking blood samples, and the killing method used is Euthanasia by leaving the fish for a period of time to die, then dissecting the fish and taking histological samples.

###  Method for calculating LC50 (Lethal concentration for 50% Mortality)

To calculate LC50, which represents the concentration of ZnO-NPs causing 50% mortality of the fish, it relies on the mortality percentages recorded at different concentrations. If the concentration causing 50% mortality is not directly observed, it is estimated through linear interpolation.

*Steps to Calculate LC50*:

1. *Calculate the mortality percentage for each concentration: *

$$\:\mathrm{Mortality\:Percentage}=\left(\frac{\mathrm{Number\:of\:Deaths}}{\mathrm{Total\:Number\:of\:Fish}}\right)\times\:100$$

2. *Determine the concentration corresponding to 50% mortality*:

Use interpolation between the concentrations where mortality transitions around 50% by formula for linear interpolation:

$$\:LC50={C}_{1}+\left(\frac{50-{P}_{1}}{{P}_{2}-{P}_{1}}\right)\times\:\left({C}_{2}-{C}_{1}\right)$$

Where: $\:{\mathrm{C}}_{1}=10\hspace{0.17em}\mathrm{mg/L}$ (lower concentration), $\:{\mathrm{C}}_{2}=20\hspace{0.17em}\mathrm{mg/L}$ (higher concentration), $\:{\mathrm{P}}_{1}=66.7\mathrm{\%}$ (mortality percentage at $\:{\mathrm{C}}_{1}$) and $\:{\mathrm{P}}_{2}=66.7\mathrm{\%}$ (mortality percentage at $\:{\mathrm{C}}_{2}$).

### Muscular tissue preparation

Fresh muscle samples (0.5 g) were homogenized in 5 mL of 0.1 M potassium phosphate buffer (pH 6.5) containing 20% glycerol, 1 mM EDTA, and 1.4 mM dithioerythritol. The homogenates were centrifuged at 3000 rpm for 5 min, and the supernatant was used for biochemical assays.

### Oxidative stress markers measurement

Reactive Oxygen Species (ROS) levels in fish flesh were determined using the oxidation method. Glutathione reduced (GSH) content was assessed, where oxidation with DTNB produced glutathione disulfide, measured at 412 nm using a spectrophotometer.

### Enzyme activity measurement

Aspartate Aminotransferase (AST) was measured using the IFCC method (1980) with Randox kits at 340 nm^22^. Alanine Aminotransferase (ALT) was measured similarly to AST using specific ALT reagents. Alkaline Phosphatase (ALP) was assayed using the method by Wright et al.^23^.

### Mineral content analysis

Fish muscle samples were dried at 180 °C for 8 h, followed by digestion with chloroform-methanol-water mixture. While, the sodium (Na), Potassium (K), and Calcium (Ca) were analyzed using Atomic Absorption Spectroscopy (AAS).

### Histological examination

To evaluate the histopathological effects of green-synthesized ZnO nanoparticles (ZnO-NPs) on liver and muscle tissues of Blue Parrotfish (*Scarus coeruleus*), tissue samples were dissected and preserved in 4% formal saline. The samples were processed following standard histological protocols as described by Assem et al.^24^. This involved dehydration through a graded ethanol series, clearing in xylene, and embedding in paraffin wax. Sections (3–5 μm thick) were obtained using a rotary microtome and stained with hematoxylin and eosin (H&E). Microscopic examination was conducted using a compound microscope, and microphotographs were captured with a Leica digital camera.

### Microbiology investigation

One ml water sample from each treated tank was collected in sterile vials and inoculated onto agar selective media for each fish pathogenic bacteria (triplicate for each). *Streptococcus* was identified using standard microbiologic methods. The characteristics used to identify streptococcal species were that they had a β-α- λ-hemolytic reaction on trypticase soy agar with 5% sheep’s blood and incubate at 28 °C for 24-48 h. *Vibrio* sp was detected using Thiosulfate Citrate Bile salts sucrose (TCBS, DM218D) agar and incubated at 28 °C for 24 h^25^. To enumerate total *Aeromonas*, one mL from each treatment was directly inoculated onto plates of selective *Aeromonas* agar medium (Bile salt orgasm brilliant green agar, LAB 167 and incubated at 28 °C for 24 h^26^.

### Compliance and ethics

Compliances with ethical standards in the experimental setup and fish handling, Fish maintenance, experimental protocols and used methods in this proposal was reviewed and approved by the Institutional Animal care and Use Committee of National Institute of Oceanography and Fisheries, Egypt. (NIOF-IACUC) under the approval code: NIOF-AQ5-F-25-R-035. and the study was reported in accordance with ARRIVE guidelines.

## Results and discussion

### Characterization of ZnO nanoparticles green synthesized

**Fig. 1:**
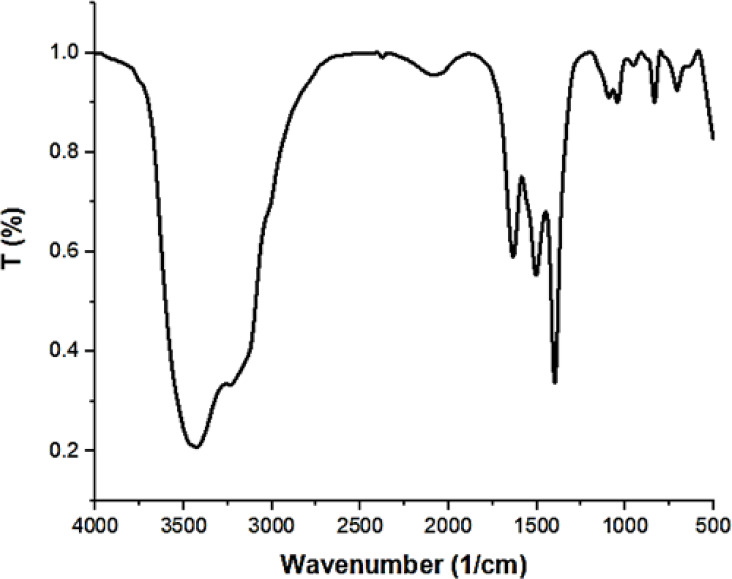
FTIR spectroscopy for ZnO-NPs.

**Fig. 2:**
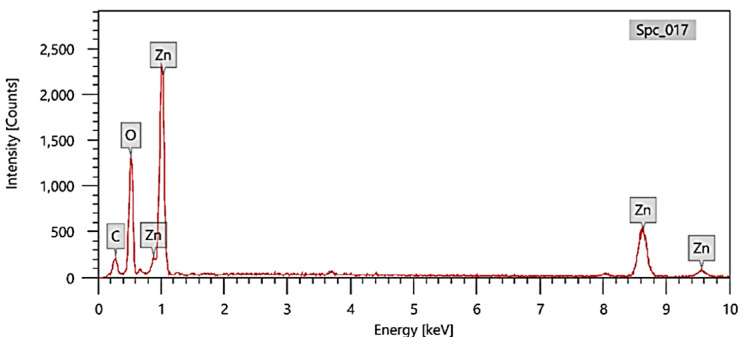
EDX analysis of ZnO-NPs.

Zeta potential is an essential parameter to be measured for the assessment of surface charge and nanoparticle colloidal stability, along with possible interactions with charged species. As evident from Fig. 1S, the green synthesized ZnO nanoparticles have a + 11.0 mV positive surface charge, which indicates that positively charged functional groups are present. Other forces such as van der Waals forces, hydrogen bonding, and dipole–dipole interactions might still permit dye uptake^27^. Zeta potential being pH-sensitive, pH alteration would possibly maximize the adsorption efficiency. Also, FTIR spectroscopy also substantiates the presence of adsorption-holding functional groups. From Fig. 1, there were typical adsorption bands ranging from 500 to 4000 cm⁻¹. The O–H and NH₂ groups could be attributed to adsorbed water or residual organic material left behind in the *Padina* algae extract. Bands related to C = C, C–H, and C–O bonds also signify the organicity of the green synthesis medium. Apparently, metal–oxygen bonds (Zn–O) also existed at 706 and 478 cm⁻¹, validating the formation of ZnO nanoparticles^28^. While, the elemental constitution analysis by Energy-Dispersive X-ray Spectroscopy (EDX), Fig. 2, revealed the presence of zinc, oxygen, and high content of carbon (10.52–21.78%). The organic stabilizers used in green synthesis are the likely cause of the carbon. Organic byproducts of carbohydrates or proteins present in the algal extract stabilize the nanoparticles molecules^29^. Functional groups such as hydroxyl or carboxyl may enhance dye adsorption through hydrogen bonding or electrostatic attraction.

**Fig. 3:**
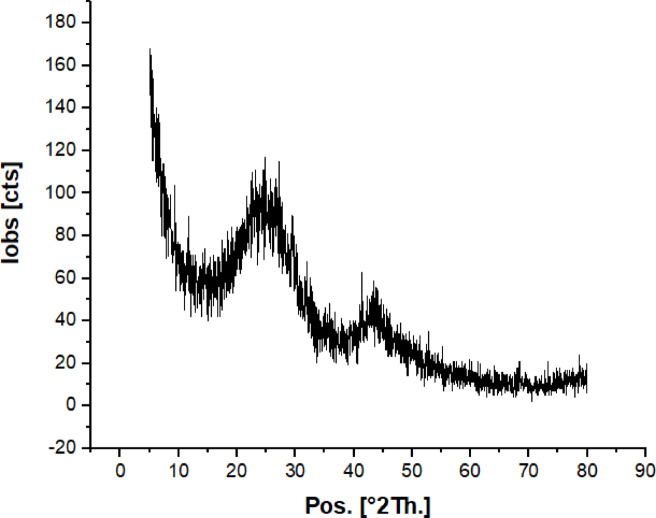
The XRD pattern of ZnO-NPs.

X-ray diffraction (XRD) was carried out to determine the crystalline structure of the ZnO nanoparticles. The diffraction peaks obtained (Fig. 3) correspond to hexagonal wurtzite form with characteristic 2θ values at 31.7°, 34.4°, 36.2°, 47.5°, 56.5°, 62.8°, and 67.7° that match with the standard JCPDS file no. 89-1397. Absence of spurious peaks validates the high purity and crystallinity of the synthesized ZnO. The crystallite size was calculated using the Debye–Scherrer formula with the strongest peak (101) and was found to be 35.89 nm. The tiny size of the crystallite signifies that there is a huge surface area-to-volume ratio, which is advantageous for uses where high reactivity is required such as photocatalysis and antimicrobial activity.

### Mortality and impact of ZnO-NPs concentrations on survival rates

As shown in Table 1, no mortality was found in the control treatment (0 mg/L), where it is confirmed that environmental conditions such as water temperature and quality were satisfactory and caused no negative effect on fish survival. Dose-dependent toxicological impact was evidenced by an increase in exposure to ZnO-NPs concentrations. For 10 mg/L, two fish died on day 11 and day 15, respectively, with a mortality of 66.7%, which is the onset of sub-acute toxicity. For 20 mg/L and 40 mg/L, identical mortality rates of 66.7% were also recorded, indicating a lasting toxic effect at moderate concentrations. Surprisingly, mortality remained constant at 66.7% even at 60 mg/L, which is contrary to the trend of increasing mortality with higher concentrations. This plateau may result from physiological acclimation, nanoparticle aggregation, and reduction in bioavailability, or individual variability in tolerance^30^. Most significantly, 100% mortality at 80 mg/L, with a sharp increase in toxicity at this dose. The 100% mortality indicates acute lethal toxicity, perhaps due to nanoparticle overaccumulation within vulnerable organs such as gills and liver, leading to instant organ failure or oxygen uptake disruption. This finding contradicts earlier conjecture of increasing toxicity at high levels due to settling particles and underscores the propensity and volatility of nanoparticle behavior in aquatic systems.

#### Effects of ZnO-NPs on fish weight and growth

For control treatment, fish registered a statistically significant increase in body weight (11.28 g) during the trial of 15 days, indicating good feeding and metabolism conditions. Growing ZnO-NPs concentrations were associated with reduced growth performance. At 10–60 mg/L concentrations, surviving fish indicated visible weight reduction or growth stagnation, likely caused by decreased appetite due to physiological stress, impaired nutrient absorption by injury of intestinal lining or liver, and metabolic disturbance due to oxidative stress. These effects are consistent with the known mechanisms of nanoparticle toxicity, such as the formation of reactive oxygen species (ROS) that damage proteins, lipids, and DNA, and disrupt normal cell function.

Interestingly, while 100% mortality at 80 mg/L, ZnO-NPs at low, sub-lethal doses are speculated to continue to serve as a nutritional supplement. Zinc is an essential micronutrient involved in enzymic function and antioxidant defense systems (e.g., superoxide dismutase). Thus, at low concentrations, ZnO-NPs may possess a dual role: useful to metabolic processes and immune modulation, yet toxic at elevated concentrations due to their nanoparticulate nature and ability to traverse biological barriers.

#### Determination of LC50 and toxicological threshold

**Fig. 4:**
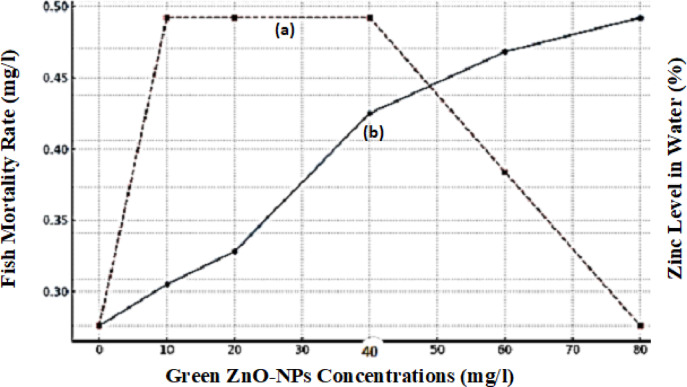
(**a**) Represents the fish mortality rate, which was high at moderate concentrations (10–40 mg/L) but decreased at the highest concentration (80 mg/L). (**b**) shows the increase in zinc levels in water with higher ZnO-NPs concentrations, indicating the gradual release of zinc ions.

The LC50 (lethal concentration to kill 50% of organisms) of ZnO-NPs was found to be 10–20 mg/L, from the pattern of death in Table 1, Fig. 4. At these concentrations, more than 50% mortality was observed in all the groups that were tested (10–60 mg/L) except for the control. This suggests that comparatively low levels of green-synthesized ZnO-NPs can cause considerable stress and death in blue parrotfish, and need to be controlled rigidly by the environment to avoid ecological harm. Furthermore, the fact that inhibition of growth preceded death at middle concentrations hints at the manifestation of sub-lethal chronic effects, and monitoring of weight becomes a critical early-warning sign for the toxicity of nanoparticles^31^.

#### Mechanistic insights into toxicity

The ZnO-NPs cytotoxicity has various mechanisms: Gill dysfunction that ZnO-NPs settle on gill surfaces, reducing respiratory fitness. ROS generation which induces oxidative stress, damaging internal organs. Zn²⁺ release by the release of ZnO-NPs elevates Zn²⁺ levels, potentially above homeostatic thresholds, in addition reduces the ability of fish to respond to environmental stressors. The nonlinear mortality pattern high mortality at low and high concentrations both may be suggestive of intricate dynamics between nanoparticle agglomeration, dissolution, and fish detoxification reactions^32^.

**Table 1:** Effect of different ZnO-NPs concentrations on mortality and growth of blue Parrotfish over 15 Days.

Concentration (mg/L)	Number of fish (Start)	Deaths after 11 days	Number of fish (End)	Total deaths (End)	Mortality (%)
0 (Control)	3	0	3	0	0
10	3	1	1	2	66.7
20	3	2	1	2	66.7
40	3	0	1	2	66.7
60	3	0	1	2	66.7
80	3	0	0	3	100

### Zinc levels in water and their relationship to the growth of blue Parrotfish

Zinc deficiency is a fence that hinders growth and metabolism in fish to influence factors such as the activation of enzymes such as carbonic anhydrase, which regulates pH and ion transport. Protein and DNA synthesis contributing to muscle and bone growth. Reinforcement of defenses against environmental stressors. Besides, improved nutrient absorption enhancing digestion and weight gain.

The data in Table 2 show the zinc levels in water at different concentrations of zinc oxide nanoparticles (ZnO-NPs). The table shows a gradual increase in dissolved zinc levels as the ZnO-NPs concentration increased. Hence, ZnO-NPs release zinc ions (Zn²⁺) into water-thus an increase in bioavailability of zinc by fish. However, the increase is not perfectly linear-means some nanoparticles may aggregate or settle with time affecting their dissolution rate^33^.

The Control Group (0 mg/L ZnO-NPs) recorded a baseline value of Zinc at 0.276 mg/L, indicating the naturally occurring zinc in water that is useful for physiological processes in fish. The highest zinc level observed was at 80 mg/L ZnO-NPs (0.492 mg/L). The increase in zinc level between this treatment and that of 60 mg/L was so modest (from 0.468 to 0.492 mg/L) even though the highest concentration of 80 mg/L of ZnO-NPs had been applied. This means the dissolution of ZnO-NPs has reached a point of saturation; therefore, adding more ZnO-NPs to the solution does not result in higher dissolved zinc concentrations. Zinc levels in water increase with higher concentrations of ZnO-NPs. This clearly indicates the gradual dissolution of ZnO-NPs, which implies the release of zinc ions (Zn²⁺) into the waters^34^.

**Table 2:** Levels of zinc in water samples.

Sample	Zinc level in water (mg/L)
Control (0 mg/L ZnO-NPs)	0.276
10 mg/L ZnO-NPs	0.305
20 mg/L ZnO-NPs	0.328
40 mg/L ZnO-NPs	0.425
60 mg/L ZnO-NPs	0.468
80 mg/L ZnO-NPs	0.492

### Zinc levels, oxidative stress, and physiological responses in *Scarus coeruleus*

Table 3 displayed the relationship between zinc levels and growth in *Scarus coeruleus* reveals a complex, dose-dependent pattern. At low to moderate ZnO-NPs concentrations (10–40 mg/L), a consistent mortality rate of 66.7% was observed. This is likely due to the rapid dissolution of ZnO-NPs and accumulation of Zn²⁺ ions in fish tissues, which disrupts ionic balance, induces oxidative stress, and alters cellular membrane function. Such conditions may destabilize metabolic processes, particularly within tetrahedrally coordinated environments like those found in biological membranes, thereby contributing to high toxicity and mortality^35^. At a concentration of 60–80 mg/L, mortality decreased to 33.3%, suggesting that fish began to mount adaptive responses. One plausible mechanism involves the upregulation of detoxifying proteins such as metallothioneins, which bind excess Zn²⁺ and mitigate its harmful effects^36^.

###  Oxidative stress and antioxidant response by biochemical assays

**Fig. 5:**
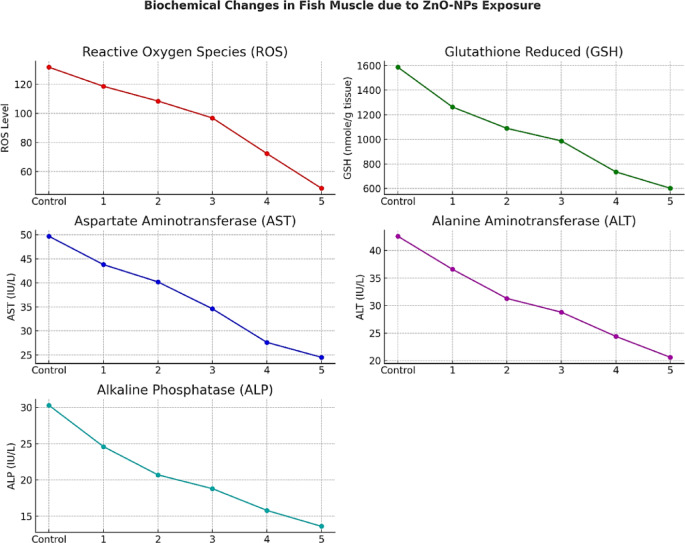
Oxidative stress markers and liver function in Blue Parrotfish tissues.

Biochemical assays revealed a non-linear oxidative stress response to ZnO-NPs. Surprisingly, the highest levels of reactive oxygen species (ROS) were recorded in the control group, ranging from 131.6 to 134.5 nmol/g tissue (Table 3 and Fig. 5). In contrast, ROS levels declined sharply at higher ZnO-NPs concentrations, reaching 44.5–51.3 nmol/g in the highest exposure group. This counterintuitive trend may reflect metabolic adaptation or reduced zinc bioavailability due to nanoparticle aggregation and slower dissolution.

However, the reduction in ROS does not necessarily indicate recovery or protection. Instead, it may correspond to cellular dysfunction where redox regulation is impaired. Supporting this, glutathione (GSH) levels a key antioxidant was significantly depleted across exposed groups, dropping from 1586.6 nmol/g in the control to approximately 589.4–615.8 nmol/g in the most affected fish^37^. This sharp decline suggests active antioxidant consumption during prolonged oxidative stress, highlighting cellular efforts to counteract ZnO-NP-induced damage.

Hepatic enzyme activity declined progressively with increasing ZnO-NPs exposure. Aspartate aminotransferase (AST) levels dropped from 49.7 to 53.3 IU/L in control fish to 23.8–26.6 IU/L in the highest exposed group. Similarly, alanine aminotransferase (ALT) decreased from 40.8 to 44.9 IU/L to 20.6–22.8 IU/L, and alkaline phosphatase (ALP) activity fell from 27.7 to 30.3 IU/L to 12.5–14.6 IU/L. These reductions suggest impaired liver function, possibly due to hepatocellular injury or suppressed metabolic activity resulting from chronic nanoparticle exposure^38^.

Such enzymatic suppression may arise from either cytotoxic inhibition or as part of a compensatory physiological adaptation to prolonged stress. Reduced enzyme levels further reinforce histological findings that showed necrosis, vacuolization, and central vein congestion in hepatic tissues across several treatment groups.

**Table 3:** Sampling and parameters measured in musculature tissues of fish.

Samples	Replicates	Parameters				
		ROS	GSH (nmole GSH/ g tissue)	AST(IU/L)	ALT (IU/L)	ALP (IU/L)
Control	1st	131.6	1586.6	49.7	42.6	30.3
	2nd	127.8	1477.4	53.3	44.9	27.7
	3rd	134.5	1563.7	51.6	40.8	29.6
1	1st	118.5	1263.5	43.8	36.6	24.6
	2nd	112.7	1232.7	41.2	35.5	23.7
	3rd	121.4	1247.9	44.5	37.4	25.5
2	1st	108.4	1089.6	40.2	31.3	20.7
	2nd	111.5	1092.8	38.8	33.6	22.6
	3rd	104.7	1071.7	39.7	30.8	21.4
3	1st	96.8	987.6	34.6	28.8	18.8
	2nd	88.4	1046.8	35.6	27.7	17.7
	3rd	97.3	994.4	33.7	30.8	19.4
4	1st	72.4	735.8	27.6	24.4	15.8
	2nd	70.7	813.5	26.5	25.3	16.7
	3rd	75.8	784.5	29.5	26.2	15.3
5	1st	48.6	602.7	24.5	20.6	13.6
	2nd	51.3	589.4	23.8	22.8	12.5
	3rd	44.5	615.8	26.6	21.6	14.6

ROS: Contents of reactive oxygen species.

Glutathione reduced (GSH) activities.

AST: Aspartate Aminotransferase.

ALT: Alanine Aminotransferase.

ALP: Alkaline Phosphatase.

### Mineral composition in muscular tissues of fish exposed to green ZnO-NPs

Analysis of mineral content in muscle tissues revealed significant disruptions in ionic balance. Levels of sodium (Na⁺), potassium (K⁺), and calcium (Ca²⁺) increased progressively with ZnO-NPs concentration. This accumulation likely stems from altered membrane permeability or nanoparticle interference with ion channels and transport proteins^39^. Such ionic disturbances can impair osmoregulatory function and neuromuscular coordination, affecting fish growth, metabolism, and behavior.

Depletion of GSH reserves, indicating prolonged antioxidant defense against oxidative injury. Suppression of liver enzyme activity (AST, ALT, ALP), signaling hepatic dysfunction were detected^40^. Disruption of ionic equilibrium, likely due to direct interaction of nanoparticles with cellular membranes and ion transport systems was studied^41^. Together, these results illustrate the dose-dependent and system-wide impacts of ZnO-NPs on fish health.

**Fig. 6:**
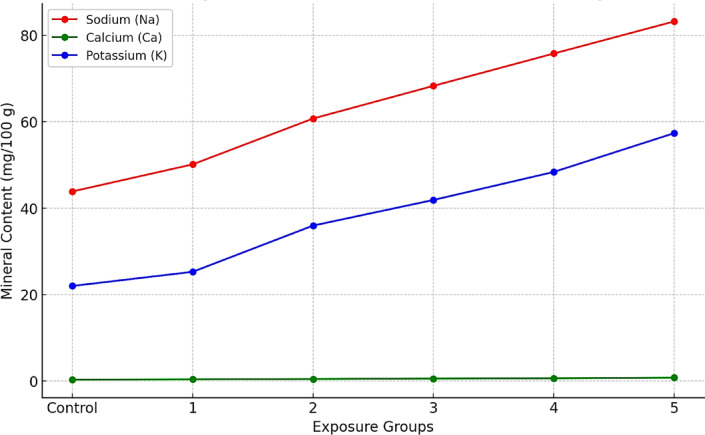
Mineral concentrations (Na, Ca, K) in fish muscle tissues after exposure to ZnO-NPs.

The mineral composition of the muscular tissues of *Blue Parrotfish* underwent a remarkable alteration following a gradual but constant exposure to green ZnO-NPs concentrations. The measured minerals Na, Ca, and K showed an increasing trend in the exposure groups compared to the control group, suggesting interference of ZnO-NPs with ion homeostasis and muscle physiology, as provided in Fig. 6.

Sodium levels increased consistently in muscle tissues of fish under higher concentrations of ZnO-NPs. The control group values were 40.09–47.43 mg/100 g, while study group 5 was 79.44–86.56 mg/100 g. Increase in Na^+^ levels may therefore be correlated with increased uptake or retention of sodium due to interaction of nanoparticles with sodium channels and transporters in muscle tissues. This may reflect an interaction of ZnO-NPs with Na^+^/K^+^ ATPase pump in the present study leading to the altered membrane permeability and thus increased sodium accumulation^42^. Raised concentrations of Na in group 5 may reflect ionic stress that might control osmoregulation and physiological activity in fish^43^.

Calcium followed control and group 5 values of 0.27–0.32 mg/100 g and 0.76–0.79 mg/100 g, respectively. Calcium plays crucial roles in the muscle contraction process, metabolic functions, and enzyme operations^44^. The increased content of calcium, possibly due to stimulated absorption or reduced excretion, could be by the contribution of ZnO-NPs on calcium transport. With the use of high concentrations of ZnO-NPs, compensatory body mechanisms can be triggered in a bid to counteract alterations demonstrated on calcium homeostasis since ZnO is also anticipated to act against calcium binding proteins and calcium signaling processes.

Potassium is a key electrolyte that is vital for muscle function and had a significant improvement with values between control group 20.21–22.31 mg/100 g and group 5 being between 54.77 and 59.68 mg/100 g. For the elevated K^+^ levels, exposure with ZnO-NPs causes effects on potassium ion channels and excitability of the muscle. The disruption of Na^+^/K^+^ homeostasis by nanoparticle exposure could potentially affect neuromuscular function that is implicated in muscle contraction and energy metabolism^45^. The maximum potassium content in group 5 shows a compensatory phenomenon where potassium is being sequestered by fish muscle tissue to offset the altered influx of sodium.

Disruption of ionic homeostasis implies that the increasing concentrations of Na, Ca, and K suggest that ZnO-NPs interfere with ion transport proteins and membrane channels which modify mineral accumulation^45^. This may have physiological implications since electrolyte imbalance would result, i.e., in case it affects muscle contraction, nerve conduction, and metabolic processes.

In fish, the development of osmoregulatory mechanisms would maintain internal ionic homeostasis. The high increase in mineral content indicates that ZnO-NPs cause osmotic stress in fish, compensating by enhanced uptake of ions or retaining ions. Prolonged disruptions would disturb cellular signaling, enzyme activity, and muscle integrity thus indicating probable metabolic stress and function depression. The increase in calcium and potassium as basic minerals in comparatively moderate amounts (groups 2–3) provides a promising indication that ZnO-NPs would prove useful as a nutritional supplement. However, increasing it beyond some extent (group 5) actually activated unduly high mineral accumulation which should be interpreted as toxicity where a threshold dosage of ZnO-NPs can become detrimental.

### Histopathological assessment of liver tissues in *Blue Parrotfish* (Scarus coeruleus) exposed to green synthesized ZnO-NPs

**Fig. 7:**
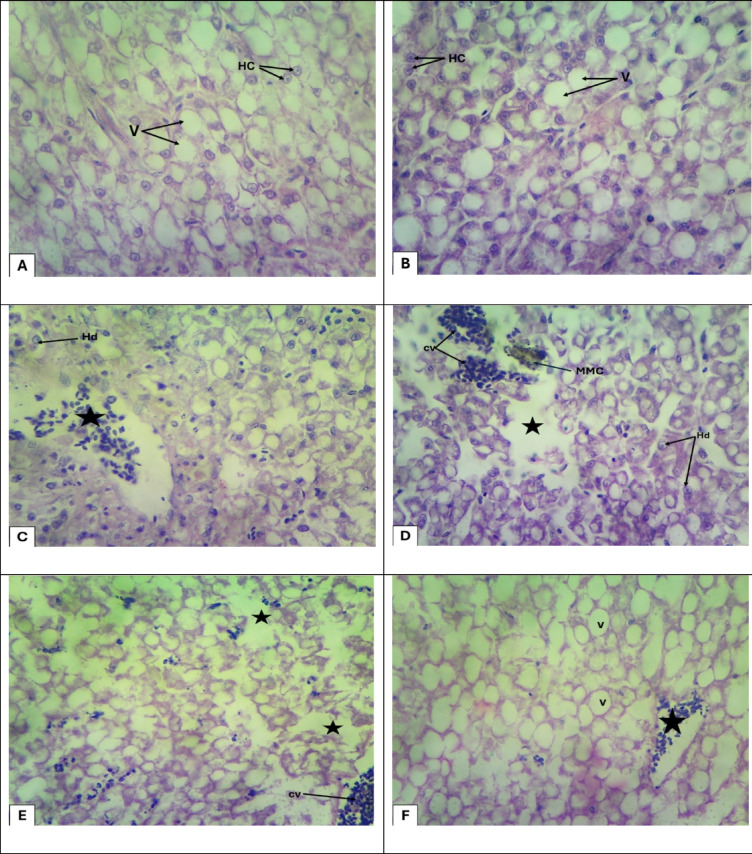
Photomicrograph liver sections of Blue Parrotfish (*Scarus coeruleus*(**A**), Control untreated fish liver tissue showing normal hepatocytes (HC). (**B**), 10 mg/L of ZnO-NPs treated fish group: showing, hepatocytes (HC) and tissue with moderate vacuoles (V). (**C**), 20 mg/L of ZnO-NPs treated fish group: showing, hydrophobic degenerated hepatocytes (Hd) and congestion of central veins (star). (**D**), 40 mg/L of ZnO-NPs treated fish group: showing, hydrophobic degenerated hepatocytes (Hd), congestion of central veins (cv), activation in Melano macrophage centers (MMC) and Necrosis (star). (**E**), 60 mg/L of ZnO-NPs treated fish group: showing, congestion of central veins (cv) and Necrosis (star). (**F**), 80 mg/L of ZnO-NPs treated fish group: showing congestion of central veins (star) and vacuoles (V) H&E. (X 400).

Control group showing normal hepatocytes with a few fat vacuoles (Fig. 7A). With increasing concentration of Green Synthesized ZnO-NPs (10, 20, 40, 60, and 80) mg/L groups: hydrophobic degenerated hepatocytes, congestion of central veins appears ZnO-NPs and it observed that the number of vacuoles was gradually increase with increasing concentration of ZnO-NPs as shown as (Fig. 7B, C, D, E and F).

In the control group, *Scarus coeruleus* muscle tissue revealed normally structured longitudinal muscle bundles, well-packed fibers, and well-formed nuclei, indicating the absence of any pathologic changes. However, with the increase in concentration of green synthesized ZnO-NPs, there were increasing changes in muscle histology. These included segmental lysis of myocytes, lymphocytic infiltration, and inter-fiber edema, as evident from the photomicrographs in Fig. 6. These structural perturbations are suggestive of increased tissue stress, inflammation, and cytotoxicity due to nanoparticle exposure.

Similarly, Fig. 7A–F illustrates the histological progression of liver damage that is linearly correlated with the level of ZnO-NPs (10–80 mg/L). These changes reflect the cumulative effects of oxidative stress and cytotoxicity on hepatocytes. In Fig. 7A, a control liver section reveals regular hepatocytes with unaltered structure and minor fat vacuolization, which reflects normal liver function under non-exposure.

In exposure to 10 mg/L ZnO-NPs (Fig. 7B), hepatocytes begin displaying modest vacuolization, reflecting the initiation of sub-lethal stress perhaps resulting from initial Zn²⁺ accumulation and slight membrane permeability alterations^46^. In 20 mg/L (Fig. 7C), hydrophobic degeneration and congestion in the central vein are now evident to reflect greater cellular injury perhaps resulting from increased oxidative stress. These findings are in concordance with biochemical observations of elevated reactive oxygen species (ROS) and deranged liver enzyme function (AST and ALT)^47^.

At 40 mg/L (Fig. 7D), there were extensive hepatocellular degeneration, extreme vascular congestion, necrosis, and MMMC activation. These histological findings suggest a strong immune response and severe tissue damage, consistent with biochemical markers of lipid peroxidation and decreased glutathione (GSH), confirming widespread oxidative damage^48^.

In Fig. 7E, group 60 mg/L, liver sections show persistent congestion and focal necrosis. Interestingly, even though exposure may be at a high level, ROS was at a lower level than at mid-range concentrations, perhaps due to adaptive responses such as metallothionein induction or enhanced activity of antioxidant enzymes. Interestingly, in 80 mg/L ZnO-NPs (Fig. 7F), liver tissue presents ongoing vascular congestion but with widespread cytoplasmic vacuolization rather than severe necrosis. This pattern reflects reduction of cytotoxicity, which may be attributed to nanoparticle agglomeration, reducing Zn²⁺ solubility and bioavailability^49^. This may explain the decreased mortality rate at this level, despite ongoing histological insult.

The histological responses are congruent with the biochemical findings of the study. Consistent lowering of liver enzyme function (ALT, AST, ALP) with increasing ZnO-NP concentration indicates compromised hepatic function. Oxidative stress biomarkers showed non-linear response with highest oxidative damage at mid-concentrations (20–40 mg/L). Moreover, increased deposition of metal ions such as sodium, calcium, and potassium in muscle tissue indicates an interference in membrane integrity and osmoregulatory balance, further confirming systemic toxicity^50^ .

Overall, these tissue-level alterations confirm an unequivocal dose-dependent toxicity of ZnO-NPs, particularly at intermediate doses at which concentrations of dissolved Zn²⁺ are sufficient to impose extreme cellular damage. Histopathological analysis of liver tissue of *Scarus coeruleus* is visible proof of nanoparticle-induced hepatotoxicity, reflecting pathological changes from vacuolization to necrosis and vascular congestion. These changes are accompanied by markers of oxidative stress and mineral imbalance, validating histopathology as an effective diagnostic tool for nanoparticle toxicity in aquatic animals. The results further underscore the necessity of ascertaining environmentally safe levels of nanoparticle release to protect aquatic environments from repeated sub-lethal toxic effects.

**Fig. 8:**
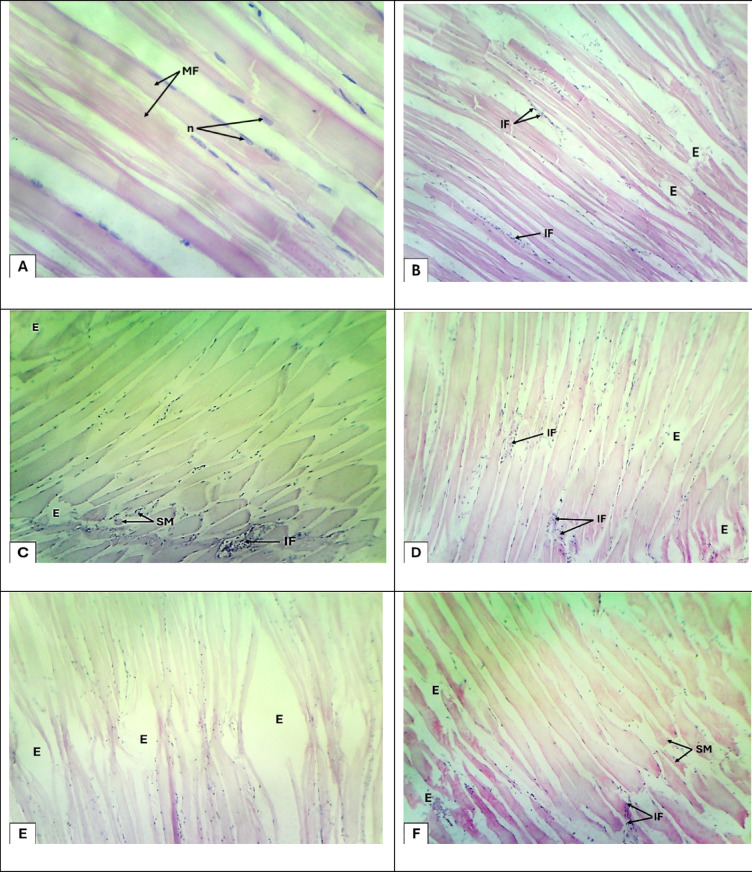
Photomicrograph muscles sections of Blue Parrotfish (*Scarus coeruleus).* (**A**), Control untreated fish showing, normal longitudinal muscle bundles with muscle fibers (MF) and nuclei (n). (**B**), 10 mg/L of ZnO-NPs treated fish group: showing, infiltration of lymphocytes (IF) and dispersed muscle bundles by edema (E). (**C**), 20 mg/L of ZnO-NPs treated fish group: showing, segmental myocytic lysis (SM), infiltration of lymphocytes (IF) and edema (E). (**D**), 40 mg/L of ZnO-NPs treated fish group: showing, infiltration of lymphocytes (IF) and edema (E). (**E**), 60 mg/L of ZnO-NPs treated fish group: showing, and dispersed muscle bundles by edema (E). (**F**), 80 mg/L of ZnO-NPs treated fish group: showing: showing, segmental myocytic lysis (SM), infiltration of lymphocytes (IF) and edema (E) H&E. (X 100).

Nonetheless, Fig. 8 presents photomicrographs of skeletal muscle sections of *Scarus coeruleus* following exposure to varying concentrations of green-synthesized zinc oxide nanoparticles (ZnO-NPs) for 15 days. Histological examination was carried out using hematoxylin and eosin (H&E) staining at 100× magnification. The photomicrographs reflect a clear, dose-related pattern of tissue injury with respect to greater concentrations of ZnO-NPs, supplementing the biochemical and physiological markers of toxicity as discussed in the study. In the control group (Fig. 8A), muscle tissue had normal histology with well-sustained muscle fibers (MF) and longitudinally oriented muscle bundles, with intact nuclei (n) centrally located. This testifies to the architectural integrity of muscle in control fish and provides a healthy baseline against which pathological alterations induced through nanoparticle exposure are measured.

At 10 mg/L ZnO-NPs (Fig. 8B), there were slight histological changes in the shape of lymphocytic infiltration (IF) and interstitial edema (E) dissociating the muscle fibers. Such minor inflammation and fluid accumulation are signs of the initial states of cellular stress, perhaps as a result of increased membrane permeability or oxidative imbalance by reaction with Zn²⁺ ions. This is in agreement with the report of the study of a moderate increase in reactive oxygen species (ROS) at this level. With treatment of 20 mg/L ZnO-NPs (Fig. 8C), the sections of muscle were more pathologically affected by segmental myocytic lysis (SM), increased lymphocytic infiltration, and greater edema. These are unequivocal signs of cytotoxic damage and inflammation, reflecting the extreme oxidative stress and low glutathione (GSH) levels reported in the biochemical assays. In addition, alterations in sodium and potassium homeostasis, also evident in the study, would have accounted for impaired muscle integrity and function under this exposure level.

In Fig. 8D (40 mg/L), muscle tissue showed chronic inflammatory infiltration and diffuse edema between the fibers. These reflect chronic oxidative stress and immune stimulation, perhaps related to augmented lipid peroxidation and continuing ionic imbalance. The observed edema is consistent with disrupted osmoregulatory balance and also reflects the systemic toxicity noted at this nanoparticle concentration^51^.

At 60 mg/L (Fig. 8E), there was general edema, which caused clear dispersion of the muscle bundles, though without major myocyte necrosis. This would suggest a shift in the direction of ionic or osmotic stress rather than direct cytolysis as the major cause of tissue damage. Such edema would result from impaired ion transport and increased membrane permeability, as reflected in the observed rise in Na⁺, K⁺, and Ca²⁺ content in muscle tissue of treated fish^50^.

Surprisingly, despite non-visibility of mortality in the 80 mg/L group (Fig. 8F), histological examination revealed re-occurrence of segmental myocytic lysis, extensive lymphocyte infiltration, and severe edema. Such findings corroborate the reality that while systemic toxicity may reduce owing to aggregation or reduced solubility of ZnO-NPs—thus avoiding Zn²⁺ ion release the muscle tissue continues to bear localized damage. Persistence of such histological lesions in the long term suggests the likelihood of sub-lethal cytotoxic effects, even when overt physiological manifestations are not apparent.

The overall histological information presented in Fig. 8 strongly confirm the toxic effect of ZnO-NPs on skeletal muscle morphology in *Scarus coeruleus*. The shift from ostensibly normal inflammation to rampant tissue destruction defined by myocyte degradation and interstitial edema is an analogous mirror image of the biochemical consequences of oxidative stress, enzyme disturbance, and mineral disturbance. By way of detail, enhanced concentrations of sodium, calcium, and potassium ions in muscle tissues attest to the dysregulation of ion flow and membrane integrity mediated by nanoparticles^52^. The sustained lymphocyte infiltration suggests active immune response to tissue injury or nanoparticle deposition, while the edema and degeneration of myocytes noted are reflections of osmotic imbalance as well as oxidative injury. Of particular interest is the maintenance of these pathological abnormalities at higher doses (e.g., 80 mg/L) with minimal mortality rates, which highlights the importance of establishing sub-lethal endpoints that can significantly affect fish behavior, mobility, feeding efficiency, and long-term survival^53^ .

In conclusion, histopathological analysis of skeletal muscle in *Scarus coeruleus* exposed to green-synthesized ZnO-NPs shows a clearly observable, dose-dependent pattern of structural degradation. This conforms to biochemical evidence on oxidative and ionic perturbation and illustrates the role of histology as a diagnostic tool in nanotoxicity studies. The muscle lesions ranging from mild inflammation to severe cytolysis underline the likelihood of chronic, sub-lethal toxicity even in the absence of mortality. This study highlights the importance of timely environmental regulations governing nanoparticle emission to safeguard aquatic life from prolonged exposure and ensuring ecological balance.

### Microbiology investigation

Table 4 exhibited that using 40 and 60 mg/ml of nanoparticles represented the good impact for the reduction in bacterial numeration or suppression of pathogenic bacteria growth. *Vibrio* sp count got higher from 18th -24th. Regardless of host developmental stage, *Vibrio* infections may occur suddenly and can lead to the loss of the entire population reared in a given aquaculture system^54^. *Aeromonas* sp was not detected throughout the experiment that is the most common bacterial pathogen *in* freshwater fish and considered a truly opportunistic pathogen, because it is relatively common in the aquaculture environment and typically does not cause disease in healthy, well-maintained fish populations^55^.

Streptococcal infection was clearly described by the 3 types of heamolytic action; the β-heamolysis showed the signification highest impact of count in the 10, 20, and 80 mg/ml nanoparticles but lower counts were observed in using 40 and 60 mgml compared with the control. Alpha and λ-hemolysis types showed a decline in count after 18th days representing the good impact of all treatment used. Fish have always been at risk of acquiring *Streptococcus* infections owing to continuous exposure and ubiquitous global presence of various bacterial strains and species^56^. Control of fish streptococcus infection mainly relies on the use of antimicrobial compounds, vaccinations, and environmental strategies^57^, of which vaccines and antimicrobial compounds have been ineffective for various reasons^58^.

**Table 4:** Fish Pathogenic Bacteria count collected from the rearing water tanks treated with green ZnO-NPs treatments along the 24 day.

Time	Treatments		Bacterial count CFU/ml				
			Vibrio	Aeromonas	Streptococcus		
					α- heamolysis	β-heamolysis	λ-heamolysis
Initial			95	0	0	250	0
Control			10	0	50	10	0
day One	1	10 mg/L	0	0	45	12	0
	2	20 mg/L	0	0	19	100	40
	3	40 mg/L	0	0	0	0	0
	4	60 mg/L	0	0	0	2	0
	5	80 mg/L	0	0	5	0	45
Control			8	0	150	75	250
14 days	1	10 mg/L	0	0	100	0	75
	2	20 mg/L	0	0	10	3	75
	3	40 mg/L	0	0	0	0	0
	4	60 mg/L	0	0	4	0	0
	5	80 mg/L	0	0	20	0	120
Control			230		0	67	0
18 days	1	10 mg/L	240	0	0	58	0
	2	20 mg/L	64	0	0	58	0
	3	40 mg/L	8	0	0	0	0
	4	60 mg/L	0	0	0	15	0
	5	80 mg/L	0	0	0	120	0
Control			250	0	0	66	0
20 days	1	10 mg/L	280	0	0	45	0
	2	20 mg/L	168	0	0	20	0
	3	40 mg/L	27	0	0	0	0
	4	60 mg/L	0	0	0	33	0
	5	80 mg/L	7	0	0	88	0
Control			280	30	0	60	0
24 days	1	10 mg/L	275	120	0	20	0
	2	20 mg/L	290	0	0	40	0
	3	40 mg/L	18	0	0	0	0
	4	60 mg/L	2	1	0	0	0
	5	80 mg/L	190	0	0	25	0

## Conclusion

This study offers robust evidence of the dose-dependent toxicological effects of greenly synthesized ZnO nanoparticles on *Scarus coeruleus*. The exposure of ZnO-NP caused intense physiological, biochemical, and histological alterations. Especially, sublethal concentrations of 10–60 mg/L activated oxidative stress, inhibited functions of liver enzymes, and altered mineral composition in muscles whereas fatal concentrations caused acute toxicity and mortality. Histological analysis has revealed progressive degeneracy in hepatic as well as muscular tissues and proves systemic toxicity. Additionally, certain concentrations (40–60 mg/L) of nanoparticles effectively inhibited pathogenic bacteria with implications for antimicrobial effects. Despite this, the pathological effects on fish health point towards the urgent need for environmental regulation of nanoparticle release by legislation. Future research needs to explore long-term sublethal effects as well as molecular mechanistic approaches of nanoparticle-induced toxicities to enable safer applications of nanotechnology in aquatic ecosystems.

## Data Availability

All data during this study are included in this article and this manuscript does not report data generation or analysis.
